# The Home-Based Older People's Exercise (HOPE) trial: study protocol for a randomised controlled trial

**DOI:** 10.1186/1745-6215-12-143

**Published:** 2011-06-08

**Authors:** Andrew Clegg, Sally Barber, John Young, Anne Forster, Steve Iliffe

**Affiliations:** 1 Academic Unit of Elderly Care & Rehabilitation, University of Leeds, Bradford Institute for Health Research, Bradford Teaching Hospitals NHS Foundation Trust, Duckworth Lane, Bradford BD9 6RJ, UK; 2 Department of Primary Care and Population Health, University College London, Rowland Hill St, London, NW3 2PF, UK

## Abstract

**Background:**

Frailty is common in older age, and is associated with important adverse health outcomes including increased risk of disability and admission to hospital or long-term care. Exercise interventions for frail older people have the potential to reduce the risk of these adverse outcomes by increasing muscle strength and improving mobility.

**Methods/Design:**

The Home-Based Older People's Exercise (HOPE) trial is a two arm, assessor blind pilot randomised controlled trial (RCT) to assess the effectiveness of a 12 week exercise intervention (the HOPE programme) designed to improve the mobility and functional abilities of frail older people living at home, compared with usual care. The primary outcome is the timed-up-and-go test (TUGT), measured at baseline and 14 weeks post-randomisation. Secondary outcomes include the Barthel Index of activities of daily living (ADL), EuroQol Group 5-Dimension Self-Report Questionnaire (EQ-5D) quality of life measure and the geriatric depression scale (GDS), measured at baseline and 14 weeks post-randomisation. We will record baseline frailty using the Edmonton Frail Scale (EFS), record falls and document muscle/joint pain. We will test the feasibility of collection of data to identify therapy resources required for delivery of the intervention.

**Discussion:**

The HOPE trial will explore and evaluate a home-based exercise intervention for frail older people. Although previous RCTs have used operationalised, non-validated methods of measuring frailty, the HOPE trial is, to our knowledge, the first RCT of an exercise intervention for frail older people that includes a validated method of frailty assessment at baseline.

**Trial registration:**

ISRCTN: ISRCTN57066881

## Background

Frailty is a common and important syndrome that is increasingly prevalent with advancing age. It is characterised by cumulative physiological decline, which results in a vulnerability to sudden changes in health status that can be triggered by relatively minor stressor events [1]. The resulting frailty phenotype includes: weight loss, exhaustion, low energy expenditure, slow gait speed and muscle weakness (sarcopenia) [2]. A recent United Kingdom (UK) study reported a frailty prevalence rate of 8.5% for women, and 4.1% for men in a population of 638 community-dwelling people aged 64-74 years [3].

Frailty is self-perpetuating; its development results in a spiral of decline that leads to worsening frailty and increased risk of adverse health consequences including disability, admission to hospital or long-term care, and death [2,4]. Because of these adverse health consequences, frailty impacts directly on health and well-being and has important health resource implications [5]. Any attenuation of the prevalence or severity of frailty is therefore likely to have large benefits for the individual, their families and for society.

Sarcopenia is characterised by loss of muscle mass and strength and is considered to be one of the key components of frailty. It is a potential target for frailty prevention interventions that incorporate exercise to increase muscle mass and strength to improve basic mobility skills such as getting up from a chair, climbing stairs and walking to the toilet. These basic mobility skills are critical for maintenance of independence in older age, and loss of these skills can lead to a requirement for increased care.

A 2009 Cochrane Review reported a synthesis of 49 randomised controlled trials (RCTs) involving older people in permanent long-term care (reasonably assumed to be a frail population) and concluded that there was good evidence that individual or group exercise programmes were acceptable and effective in improving mobility and other daily living tasks in this vulnerable population [6].

However, the large majority of older people in the UK live at home [7]. Exercise interventions for older people living at home can be delivered either individually in their homes, or elsewhere as a group activity. A 2005 Cochrane review concluded that both home-based and group-based exercise interventions are associated with improved outcomes for patients receiving cardiac rehabilitation, but that home-based interventions may be associated with improved adherence [8]. Furthermore, the 2006 UK Department of Health (DH) white paper 'Our health, our care, our say: a new direction for community services' argued for a different, more community-based approach to people with long term conditions with the provision of more and better quality services summarized as "care closer to home" [9]. A successful home-based exercise intervention for frail older people would have the potential to improve the health and well-being in this vulnerable group at high risk of important adverse health outcomes which have substantial health and socioeconomic costs.

A home-based exercise intervention for frail older people is an example of a complex health intervention. The Medical Research Council (MRC) framework for the development and evaluation of complex health interventions identifies the key elements of the design and evaluation process [10]. We have therefore adopted the MRC framework to design and evaluate the Home-Based Older People's Exercise (HOPE) programme, an exercise programme to improve the mobility and daily living activities of frail older people living at home. This report describes the methodology of the HOPE trial; a pilot randomised controlled clinical trial to test the effectiveness of the HOPE programme. This report also describes the development of the HOPE programme with reference to the MRC framework.

## Objectives

To conduct a pilot RCT to:

1) explore the feasibility of identification of frail older people in community settings;

2) assess the acceptability of the HOPE programme to frail older people;

3) test for a preliminary estimate of effectiveness;

4) test the feasibility of recording data to identify the therapy resources required to deliver the HOPE programme;

5) gather data to inform the design of a definitive clinical trial

## Methods/Design

The HOPE trial design and methodology is based on the MRC framework [10] and international guidelines for the design of RCTs of interventions to prevent functional decline and disability in frail older people [11].

The HOPE trial is a two arm, assessor blind pilot RCT to assess the effectiveness of an exercise intervention (the HOPE programme) designed to improve the mobility and functional abilities of frail older people living at home, compared with usual care. The 12 week HOPE programme is delivered to trial participants by community-based therapists from Bradford Teaching Hospitals NHS Foundation Trust. Follow-up is at 14 weeks post randomisation.

### Setting

Bradford, UK; a post-industrial ethnically diverse northern city with a population of 350,000 people.

### Inclusion criteria

Those eligible for inclusion are frail older people. A research question under investigation in this pilot trial is to identify the most efficient recruitment method(s) for this hard to reach group of people. We have therefore used multiple recruitment sources to identify frail older people:

1. Older people living at home and under the care of a case manager (CM) in Bradford, UK. CMs are experienced nurses specifically trained in the management of long term conditions and provide nurse-led case management working alongside primary care teams and social service staff. The case management service has been developed nationally with a specific policy objective of reducing acute hospital admissions for people with multiple long-term conditions, who are more likely to be frail. There are a number of models of case management within the Bradford Primary Care Trust (PCT), including case management by Community Matrons and Advanced Nurse Practitioners. CMs have been identified with the assistance of the NHS Bradford & Airedale General Services Management Team following a series of educational meetings.

2. Older people who are housebound, defined as being unable to leave the house without the assistance of another person. Housebound older people are identified through Read code searching of general practitioner (GP) registers of National Institute for Health Research (NIHR) 'Research Ready' GP practices in Bradford, UK; social services sites providing day centre and respite care in Bradford, UK; at discharge from intermediate care hospitals in Bradford, UK; and elderly medicine outpatient clinics at Bradford Teaching Hospitals National Health Service (NHS) Foundation Trust.

The initial method of approach for those identified through GP registers of NIHR Research Ready practices is by letter from the GP practice. The initial method of approach for all other potential participants is by face-to-face or telephone contact from the healthcare professional or senior member of social services staff who is co-ordinating care.

### Exclusion criteria

1. Unable to stand and walk independently

2. Current participation in an alternative exercise programme (e.g. falls prevention programme, pulmonary rehabilitation)

3. Registered blind

4. Poorly controlled angina

5. Another member of the household already in the HOPE trial

6. Severe dementia

7. Receiving palliative care

### Development of the HOPE programme

The development of the HOPE programme has followed a three-stage process that has been informed by the MRC framework [10]. Firstly, international guidelines on the development of exercise interventions for older people from the American College of Sports Medicine (ACSM) and the American Heart Association (AHA) were reviewed [12]. These guidelines recommend that exercise interventions for older people should be multi-dimensional and include exercises to improve whole body muscle strength, mobility, balance and aerobic capacity.

Secondly, a systematic review of the literature was conducted to identify RCTs of high methodological quality that investigated home-based exercise interventions for frail older people. This review was supported by an earlier systematic review of exercise interventions for frail older people in long-term care [13]. The key components of the exercise interventions in the successful RCTs of high methodological quality were then identified to inform the frequency, intensity and duration of the intervention.

Thirdly, a series of multiperspective focus group meetings with frail older people and experienced healthcare professionals were conducted. The meetings were audio-recorded and transcribed to enable a thematic analysis of the qualitative data using grounded theory methods. The key aims and objectives of these focus group meeting were to identify key physical limitations and difficulties with activities of daily living (ADL) in frail older people living at home. This was to enable the selection of appropriate exercises to address these limitations and difficulties. The key physical limitations identified from the thematic analysis were; muscle weakness; pain and stiffness in joints; poor balance; poor postural stability and flexibility; and breathlessness. Important difficulties in ADL were standing up from a chair; getting out of bed and climbing stairs. Although aerobic exercise for older people is recommended in the ACSM and AHA guidelines, the multiperspective focus group meetings identified that aerobic exercise for frail older people would be difficult to achieve, particularly in those who are most frail because of limits in energy expenditure.

Following this three stage process, a draft intervention was produced. The draft intervention was further refined by expert consensus using Delphi methodology [14] to produce the HOPE programme - an evidence based, user defined exercise intervention for frail older people living at home.

### Description of the intervention

The HOPE programme is a 12 week progressive exercise intervention which is presented to participants in an exercise manual and delivered by trained community-based physiotherapists from Bradford Teaching Hospitals NHS Foundation Trust. The manual contains five sections; 1) information, 2) safety tips, 3) good posture, 4) exercises and 5) staying on track. The exercises have been selected to target the physical limitations and ADL difficulties identified by the focus group meetings and refined through the consensus process. To account for the diversity of our participant group, the HOPE programme is graded into three levels. Participants are stratified to the appropriate level using the baseline timed-up and go test [15]. All of the exercises are easy to learn, require no special equipment and can be performed without professional supervision. The exercises for each level of the programme (Level 1, 2 and 3), their purpose (to improve strength, mobility, balance or aerobic capacity) and their functional relevance (e.g. to improve standing up from a chair) are provided in Additional file 1, appendix 1.

At the beginning of the intervention participants are requested to perform five repetitions of each exercise in the routine. This progresses to 10 and then 15 repetitions as performance improves. The exercise routine takes less than 15 minutes to complete, and participants are requested to complete the routine 3 times a day on 5 days of the week.

**Figure 1:**
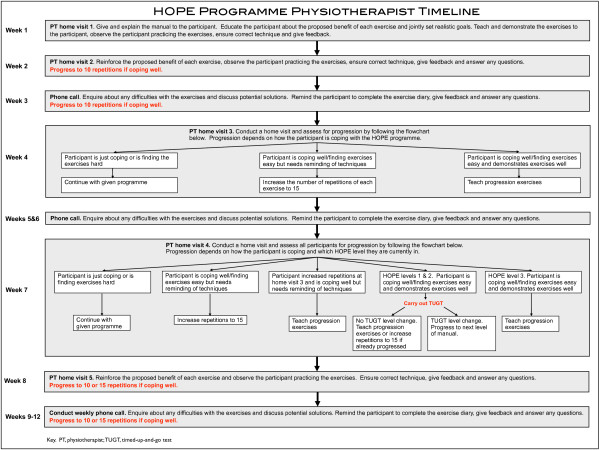
**HOPE programme physiotherapist timeline**. Copyright ^© ^2011.

Participants receive weekly support from physiotherapists through either a face-to-face home visit or telephone call (Figure 1). If participants are coping well with the exercises they are encouraged to progress within the programme. Progression is by increasing repetitions, introducing new exercises or advancing to the next level of the HOPE programme (Figure 1). Gentle aerobic exercise is incorporated as a progression exercise at Level 3 of the HOPE programme to introduce the concept of aerobic exercise to the more physically able participants.

### Strategies to support behaviour change

The HOPE programme is based predominantly on the social cognitive model of behaviour change as a theoretical framework, and this has previously been shown to successfully increase physical activity participation in adults [16-19]. Details of the behaviour change techniques and how they are incorporated into the HOPE programme are shown in Table 1.

**Table 1:** Behaviour change techniques used in the HOPE programme.

Behaviour change technique (theoretical framework)	How the technique is in the intervention
Provide information on consequences (SCogT)	Information given about the value of exercise for health in older age

Provide general encouragement (SCogT)	Praise and encouragement given by physiotherapists weekly during either a home visit or telephone call

Set graded tasks (SCogT)	Opportunities to progress (increased repetitions, addition of progression exercises, advancing to the next level of the programme) are discussed between participant and physiotherapists and an individual progression plan is agreed upon

Provide instruction (SCogT)	Each participant receives a HOPE programme manual which gives instructions for the programme and describes in written and pictorial format how to perform each exercise. Physiotherapists also provide instruction during home visits and telephone calls

Model or demonstrate the behaviour (SCogT)	Physiotherapists demonstrate how to correctly perform exercises during home visits

Prompt specific goal setting (CT)	The physiotherapists facilitates the participant to set specific functional improvement goals

Prompt self-monitoring behaviour (CT)	The participant is asked to keep a record of which days they do their exercises on and how many times they complete the routine that day

Prompt practice (OC)	Participants are provided with a HOPE programme fridge magnet to prompt them to perform their exercise routine

Relapse prevention	3 "gentle exercises" are provided in the HOPE manual for participants to complete if they are "having a bad day" (this was identified in focus groups as a situation likely to result in failure to maintain the exercise programme)

Techniques and theoretical frameworks were defined using [16]. SCogT, social cognitive theory; CT, control theory; OC, operant conditioning.

### Sample size

A successful pilot RCT provides important information regarding process, resources, management and scientific data [20]. This pilot RCT is designed to explore:

1) Process: test feasibility of the trial and intervention e.g. trial recruitment rates, drop out rates, intervention compliance

2) Resources: record resources required to identify and gain consent from participants, time to complete study assessments, resources required to deliver the intervention

3) Management: gain insight into trial personnel and data management issues

4) Scientific: estimate intervention effect size and variance, test intervention safety

To provide useful process, resources, management and scientific data we aim to recruit 100 participants (50 per group). The results from our pilot RCT will inform the feasibility, design and power calculation for a future definitive RCT.

### Randomisation

Participants undergo central, concealed randomised allocation to intervention or usual care. Participants are stratified by the baseline timed up and go test and undergo randomisation using restricted blocks of random size with an allocation ratio of 1:1. Generation and storage of the HOPE trial randomisation sequence, and conduction of individual participant randomisation is conducted by the University of Leeds Clinical Trials Research Unit (CTRU), ensuring allocation concealment.

### Baseline Assessment

Baseline assessment is conducted by an elderly care researcher and includes age, sex, cognitive assessment, co-morbidity index [21] and the Edmonton Frail Scale (EFS), a validated and reliable measure of frailty [22]. The EFS samples 10 domains, including cognitive impairment, functional ability and mobility. The maximum score is 17, which represents the highest level of frailty. The EFS demonstrates good inter-rater reliability (estimated κ = 0.7) and internal consistency (Cronbach's α = 0.62).

Involvement in previous formal exercise programmes and the dates of any previous involvement are also recorded.

### Primary outcome measure

1. Timed Up and Go test (TUGT) [15], measured at baseline and 14 weeks post-randomisation. The TUGT measures, in seconds, the time taken to stand up from a standard chair, walk a distance of 3 metres, turn, walk back to the chair and sit down. A chair with armrests and a seating depth of 44-47 cm is recommended. Those who complete the test in less than 20 seconds tend to be independently mobile, able to get in and out of a chair without assistance and climb stairs. Those who complete the test in 30 seconds or more tend to require assistance with getting in and out of a chair, climbing stairs and leaving the house. Those who complete the test in 20-29 seconds demonstrate greater variability in mobility, balance and functional ability [15]. The TUGT score demonstrates high inter-rater and intra-rater reliability (intraclass correlation coefficients (ICCs) 0.99 and 0.99 respectively) and correlates well with measures of gait speed, functional ability and balance. An improvement of 1.4 seconds on the TUGT (within-patient change score) has been identified as the minimum clinically important difference (MCID) [23].

### Secondary outcome measures

1. Barthel Index of ADL [24], measured at baseline and at 14 weeks post-randomisation. The Barthel Index assesses functional status on a 20 point scale by recording ability to complete ten activities of daily living; bathing, bladder function, bowel function, dressing, feeding, grooming, mobility, stairs, toilet use and transfers. Higher scores indicate greater independence. An MCID of 1.85 points has been identified, but the utility of the Barthel Index can be limited by floor and ceiling effects [25,26].

2. The EuroQol Group 5-Dimension Self-Report Questionnaire (EQ-5D) [27], measured at baseline and at 14 weeks post-randomisation. The EQ-5D is a standardised measure of health utility (quality of life) comprising five dimensions: mobility, self-care, usual activities, pain/discomfort and anxiety/depression. Each dimension has three levels: no problems, some problems, severe problems. The scores for each of the five dimensions are combined in a five digit number representing health status that can be converted into a summary index (0 for dead, 1 for perfect health and negative values for states worse than death).

3. Geriatric Depression Scale (GDS) [28], measured at baseline and at 14 weeks post-randomisation. The GDS is a screen for the presence and severity of depression in older people. It comprises 15 individual questions; a score of 0-4 indicates no depression, 5-10 is suggestive of mild depression and 11+ is suggestive of severe depression.

We are also recording recruitment rates; proportions and reasons for non-consent; protocol compliance by therapists; rates of adherence to the HOPE programme by patients; drop out rates; rates of completion of outcome measures; unscheduled admissions to hospital. We also record falls and document muscle/joint pain.

We anticipate that the main cost of the intervention will be the future therapy resources associated with delivery of the HOPE programme. To perform a formal cost-effectiveness analysis in the future definitive trial, we will therefore test the feasibility of collection of data to identify the therapy resources required for delivery. This data will be recorded by the physiotherapists who deliver the intervention and will include time taken for each physiotherapist home visit, time for telephone consultations and travel time.

### Analysis plan

The exploratory nature of this pilot study requires mainly descriptive statistics and will focus on the estimation of effects sizes, 95% confidence intervals and variation estimation for planning of the future definitive trial. We will compare the baseline differences between the control and intervention groups in terms of the baseline assessment tests and assess differences in any other potential confounding variables (age, sex, co-morbidity).

All outcome measures will be summarised and 95% confidence intervals constructed for the difference in outcomes between control and intervention groups. Analysis of covariance (ANCOVA) tests will be used for continuous outcomes, with adjustment for baseline values. Both adjusted and unadjusted values will be reported to detect which has the smaller variance for future planning. Risk ratios with 95% confidence intervals will be used for binary outcomes and rate ratios with resulting 95% confidence intervals will be used to assess rates.

The final intention-to-treat analysis will include all randomised participants for whom the follow-up assessment of the primary outcome measure is available. The per-protocol analysis will include all randomised participants who are deemed to have no protocol violations.

### Protocol violations

Participants who are randomised to the HOPE programme (intervention) arm but do not undertake any of the HOPE programme are deemed to be protocol violations.

### Risks

Safe exercise guidelines are followed and exercise intensity is increased gradually by therapists with experience in the delivery of exercise interventions to frail older people living at home. All potential participants are informed of the potential risks in the participant information documents provided, so that informed consent is taken with full knowledge of potential risks.

### Adverse events

An adverse event (AE) is any unfavourable and unintended sign, symptom, syndrome or illness that develops or worsens during the period of observation in the trial. This includes:

1. Exacerbation of a pre-existing illness

2. Increase in frequency or intensity of a pre-existing episodic event or condition

3. Condition detected or diagnosed after the intervention even though it may have been present prior to the start of the trial

4. Continuous persistent disease or symptoms present at baseline that worsen following the start of the trial.

A serious adverse event (SAE) is any AE occurring following trial mandated procedures that results in any of the following outcomes:

1. Death

2. A life-threatening adverse event

3. Inpatient hospitalisation

4. A disability/incapacity

All AEs are assessed for seriousness, causality and expectedness by the trial chief investigator and recorded and closely monitored. The chief investigator is informed immediately following any SAE to determine causality and expectedness. Any SAE that is deemed to be directly related to the trial intervention, or suspected to be related to the trial intervention, will be reported immediately to the ethics committee.

### Informed consent

Informed, written consent is obtained from all trial participants by an elderly care researcher during a visit to the participant's home. Consent is in full accordance with the Mental Capacity Act 2005 [29] and International Conference on Harmonisation-Good Clinical Practice (ICH-GCP) [30]. It is emphasised that participants are free to withdraw from the trial at any time and that this will not affect the future care that they receive.

### Ethical and organisational review

Ethical approval for the HOPE trial has been granted by the Bradford Research Ethics Committee (application number 09/H1302/55). NHS Research & Development (R&D) approval has been granted by NHS Bradford and Airedale and Bradford Teaching Hospitals NHS Foundation Trust.

## Discussion

The HOPE trial is a pilot RCT of an exercise intervention designed to improve the mobility and functional abilities of frail older people living at home. Although previous RCTs have used operationalised, non-validated methods of measuring frailty, the HOPE trial is, to our knowledge, the first RCT of an exercise intervention for frail older people that includes a validated method of frailty assessment at baseline.

The main challenges that we anticipate are the recruitment and retention of trial participants who are frail older people at increased risk of adverse outcomes, including falls, admission to hospital and death. We aim to maximise participant retention by regular therapist face-to-face and telephone contact. Previous trials have reported an increase in participant falls risk following exercise intervention [31]. We aim to minimise this risk through the delivery of our graded exercise intervention by therapy staff who are fully trained and experienced in the complex risk assessment techniques that are required for frail older people.

The findings from the pilot HOPE trial will inform the design and development of a future definitive multi-site RCT and guide the future commissioning of local and national therapy services for frail older people.

## Trial registration and date of first participant randomisation

Current Controlled Trials - International Standard Randomised Controlled Trial Number ISRCTN57066881. Date of trial registration 19/05/2010. Date of first participant randomisation 15/07/2010.

## List of abbreviations

ACSM: American College of Sports Medicine; ADL: activities of daily living; AE: adverse event; AHA: American Heart Association; ANCOVA: analysis of covariance; CM: case manager; CTRU: Clinical Trials Research Unit; DH: Department of Health; EFS: Edmonton Frail Scale; EQ-5D: EuroQol Group 5-Dimension Self-Report Questionnaire; GDS: Geriatric Depression Scale; GP: general practitioner; HOPE: Home-Based Older Peoples Exercise; ICC: intra-class correlation coefficient; ICH-GCP: International Conference on Harmonisation - Good Clinical Practice; ISRCTN: International Standard Randomised Controlled Trial Number; MCID: minimum clinically important difference; MRC: Medical Research Council; NHS: National Health Service; NIHR: National Institute for Health Research; PCT: Primary Care Trust; RCT: randomised controlled trial; R&D: research & development; SAE: serious adverse event; TUGT: timed-up-and-go test; UK: United Kingdom

## Competing interests

The authors declare that they have no competing interests.

## Authors' contributions

All authors contributed to the development and writing of the trial protocol. All authors read and approved the final manuscript

## Supplementary Material

Additional file 1 **Appendix 1. HOPE programme exercises**. Exercises included in the three levels of the HOPE programme. P, progression exercise; * may also reduce arthritic pain at the mobilized joint. Copyright ^© ^2011. We confirm that signed consent has been obtained from the two HOPE manual models for publication of the photographs included in Appendix 1. Click here for file

## References

[B1] Walston J Hadley EC Ferrucci L Guralnik JM Newman AB Studenski SA Ershler WB Harris T Fried LP Research agenda for frailty in older adults: toward a better understanding of physiology and etiology: summary from the American Geriatrics Society/National Institute on Aging Research Conference on Frailty in Older Adults J Am Geriatr Soc 2006 54 6 991 1001 10.1111/j.1532-5415.2006.00745.x 16776798

[B2] Fried LP Tangen CM Walston J Newman AB Hirsch C Gottdiener J Seeman T Tracy R Kop WJ Burke G McBurnie MA Frailty in older adults: evidence for a phenotype J Gerontol A Biol Sci Med Sci 2001 56 3 M146 56 11253156 10.1093/gerona/56.3.m146

[B3] Syddall H Roberts HC Evandrou M Cooper C Bergman H Aihie Sayer A Prevalence and correlates of frailty among community-dwelling older men and women Age Ageing 2010 39 2 197 203 10.1093/ageing/afp204 20007127 PMC3546311

[B4] Rockwood K Song X MacKnight C Bergman H Hogan DB McDowell I Mitnitski A A global clinical measure of fitness and frailty in elderly people CMAJ 2005 173 5 489 95 16129869 10.1503/cmaj.050051 PMC1188185

[B5] Poole T Funding adult social care in England 2009 The Kings Fund; March 1 11

[B6] Forster A Lambley R Hardy J Young J Smith J Green J Burns E Rehabilitation for older people in long-term care. Cochrane database of systematic reviews 2009 1 CD004294 10.1002/14651858.CD004294.pub2 19160233

[B7] Department for Work and Pensions Focus on Older People 2004 London: Office for National Statistics 2001

[B8] Ashworth NL Chad KE Harrison EL Reeder BA Marshall SC Home versus center based physical activity programs in older adults Cochrane Database Syst Rev 2005 1 CD004017 10.1002/14651858.CD004017.pub2 PMC6464851 15674925

[B9] Our health, our care, our say: a new direction for community services 2006 Department of Health, London

[B10] Medical Research Council Developing and evaluating complex interventions: new guidance 2008 London: MRC

[B11] Ferrucci L Guralnik JM Studenski S Fried LP Cutler GB Jr Walston JD Designing randomized, controlled trials aimed at preventing or delaying functional decline and disability in frail, older persons: a consensus report J Am Geriatr Soc 2004 52 4 625 34 10.1111/j.1532-5415.2004.52174.x 15066083

[B12] Nelson ME Rejeski WJ Blair SN Duncan PW Judge JO King AC Macera CA Castaneda-Sceppa C Physical activity and public health in older adults: recommendation from the American College of Sports Medicine and the American Heart Association Med Sci Sports Exerc 2007 39 8 1435 45 10.1249/mss.0b013e3180616aa2 17762378

[B13] Forster A Lambley R Young JB Is physical rehabilitation for older people in long-term care effective? Findings from a systematic review Age Ageing 2010 39 2 169 75 10.1093/ageing/afp247 20097661

[B14] Linstone H Turoff M The Delphi method: techniques and applications 1975 Reading, MA: Adison-Wesley

[B15] Podsiadlo D Richardson S The timed "Up & Go": a test of basic functional mobility for frail elderly persons J Am Geriatr Soc 1991 39 2 142 8 1991946 10.1111/j.1532-5415.1991.tb01616.x

[B16] Abraham C Michie S A taxonomy of behavior change techniques used in interventions Health Psychol 2008 27 3 379 87 18624603 10.1037/0278-6133.27.3.379

[B17] Baker G Gray SR Wright A Fitzsimons C Nimmo M Lowry R Mutrie N The effect of a pedometer-based community walking intervention "Walking for Wellbeing in the West" on physical activity levels and health outcomes: a 12-week randomized controlled trial Int J Behav Nutr Phys Act 2008 5 44 18775062 10.1186/1479-5868-5-44 PMC2546435

[B18] Hughes AR Gillies F Kirk AF Mutrie N Hillis WS MacIntyre PD Exercise consultation improves short-term adherence to exercise during phase IV cardiac rehabilitation: a randomized, controlled trial J Cardiopulm Rehabil 2002 22 6 421 5 10.1097/00008483-200211000-00007 12464830

[B19] Kirk A Mutrie N MacIntyre P Fisher M Effects of a 12-month physical activity counselling intervention on glycaemic control and on the status of cardiovascular risk factors in people with Type 2 diabetes Diabetologia 2004 47 5 821 32 10.1007/s00125-004-1396-5 15138687

[B20] Thabane L Ma J Chu R Cheng J Ismaila A Rios LP Robson R Thabane M Giangregorio L Goldsmith CH A tutorial on pilot studies: the what, why and how BMC Med Res Methodol 2010 10 1 10.1186/1471-2288-10-1 20053272 PMC2824145

[B21] Charlson M Szatrowski TP Peterson J Gold J Validation of a combined comorbidity index J Clin Epidemiol 1994 47 11 1245 51 10.1016/0895-4356(94)90129-5 7722560

[B22] Rolfson DB Majumdar SR Tsuyuki RT Tahir A Rockwood K Validity and reliability of the Edmonton Frail Scale Age Ageing 2006 35 5 526 9 10.1093/ageing/afl041 16757522 PMC5955195

[B23] Wright AA Cook CE Baxter GD Dockerty JD Abbott JH A comparison of 3 methodological approaches to defining major clinically important improvement of 4 performance measures in patients with hip osteoarthritis J Orthop Sports Phys Ther 41 5 319 27 10.2519/jospt.2011.3515 21335930

[B24] Mahoney FI Barthel DW Functional Evaluation: The Barthel Index Md State Med J 1965 14 61 5 14258950

[B25] Hsieh YW Wang CH Wu SC Chen PC Sheu CF Hsieh CL Establishing the minimal clinically important difference of the Barthel Index in stroke patients Neurorehabil Neural Repair 2007 21 3 233 8 10.1177/1545968306294729 17351082

[B26] Wellwood I Dennis MS Warlow CP A comparison of the Barthel Index and the OPCS disability instrument used to measure outcome after acute stroke Age Ageing 1995 24 1 54 7 10.1093/ageing/24.1.54 7762463

[B27] The EuroQol Group EuroQol-a new facility for the measurement of health-related quality of life Health Policy 1990 16 3 199 208 10109801 10.1016/0168-8510(90)90421-9

[B28] Sheikh JI Yesavage JA Brooks JO Friedman L Gratzinger P Hill RD Zadeik A Crook T Proposed factor structure of the Geriatric Depression Scale Int Psychogeriatr 1991 3 1 23 8 10.1017/S1041610291000480 1863703

[B29] Department for Constitutional Affairs Mental Capacity Act 2005 Code of Practice 2007 TSO (The Stationery Office), London

[B30] International Committee on Harmonisation Good Clinical Practice (ICH-GCP) Guidelines 2005 http://www.ich.org/products/guidelines.html

[B31] Lawton BA Rose SB Elley CR Dowell AC Fenton A Moyes SA Exercise on prescription for women aged 40-74 recruited through primary care: two year randomised controlled trial BMJ 2008 337 a2509 10.1136/bmj.a2509 19074218 PMC2769033

