# Supplementary material for: The Home-Based Older People's Exercise (HOPE) trial: study protocol for a randomised controlled trial
Source: Trials. 2011 Jun 8;12:143. doi: 10.1186/1745-6215-12-143 (PMC3121609; doi:10.1186/1745-6215-12-143)
Supplement: Additional file 1 — Appendix 1. HOPE programme exercises. Exercises included in the three levels of the HOPE programme. P, progression exercise; * may also reduce arthritic pain at the mobilized joint. Copyright © 2011. We confirm that signed consent has been obtained from the two HOPE manual models for publication of the photographs included in Appendix 1. [file 1745-6215-12-143-S1.DOC]

**Appendix 1. HOPE programme exercises**

| **Exercise** | | **Level** | **Purpose** | **Functional relevance** |
| --- | --- | --- | --- | --- |
| Breathing warm up | 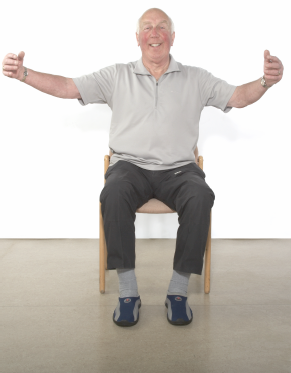 | 1, 2, 3 | A preparatory exercise to increase lung capacity | Reduce shortness of breath |
| Sitting-up | 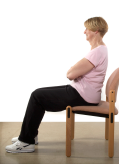 | 1, 2, 3 | Trunk/abdominal strength | Stair climbing, standing up from a chair, walking, balance |
| Spine rotation | 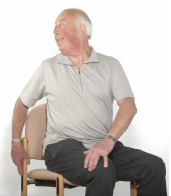 | 1, 2, 3 | Trunk mobility | Washing and dressing, reaching for something on a shelf, stair climbing |
| Armchair rise – arm strength | 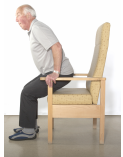 | 1 | Upper body strength | Standing up from a chair, lifting/carrying household objects |
| Leg kicks | 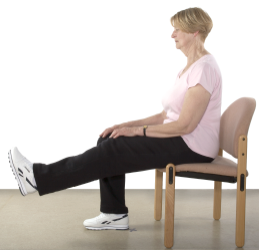 | 1 | Lower body strength | Standing up from a chair, walking * |
| Toe-heel pointing | 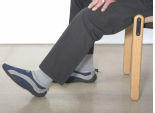 | 1 | Lower body strength | Walking, stair climbing * |
| Marching | 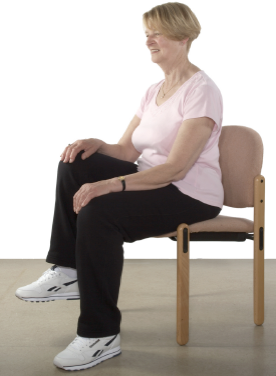 | 1P, 2 | Lower body strength | Standing from a chair, stair climbing * |
| Standing arm raises (for 30 seconds) | 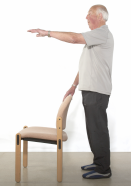 | 1P | Upper body strength and mobility and balance | Reaching for something on a shelf, lifting/carrying household objects, washing and dressing falls prevention |
| Armchair rise – arm and leg strength | 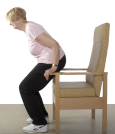 | 2 | Upper and lower body strength | Walking, stair climbing, standing up from a chair, lifting/carrying household objects |
| Calf raises | 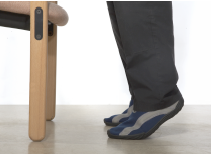 | 2 | Lower body strength and balance | Walking and stair climbing |
| Leg swing back | 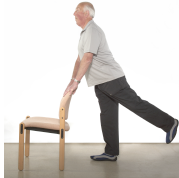 | 2P | Lower body strength and hip mobility | Standing up from a chair, dressing* |
| Side stepping | 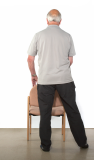 | 2P | Balance | Falls prevention |
| Chair rise | 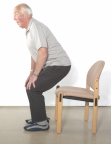 | 3 | Lower body strength | Walking, stair climbing, standing up from a chair |
| Wall press-up | 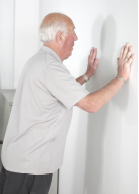 | 3 | Upper body strength and mobility | Lifting/carrying household objects, washing and dressing |
| Single foot calf raise | 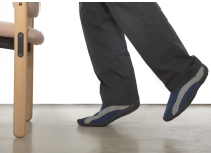 | 3 | Lower body strength | Walking and stair climbing |
| Leg back swing and side raise | 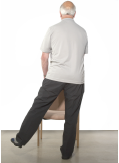 | 3P | Lower body strength and hip mobility | Standing from a chair, dressing * |
| Stand on one leg | 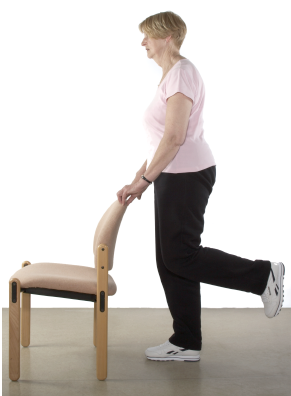 | 3P | Balance | Falls prevention |
| Walking toe to heel | 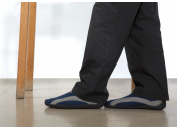 | 3P | Balance | Falls prevention |
| Aerobic exercises | 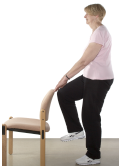 | 3P | Aerobic | Sustaining engagement in physical activities * |

Appendix 1. Exercises included in the three levels of the HOPE programme. P, progression exercise; * may also reduce arthritic pain at the mobilized joint. Copyright © 2011.
